# Phase II study of gemcitabine and cisplatin in locally advanced/metastatic oesophageal cancer

**DOI:** 10.1038/sj.bjc.6602842

**Published:** 2005-11-08

**Authors:** J Millar, P Scullin, A Morrison, B McClory, L Wall, D Cameron, H Philips, A Price, D Dunlop, M Eatock

**Affiliations:** 1 Northern Ireland Cancer Clinical Trials Unit, Belfast City Hospital, Lisburn Road, Belfast BT9 7AB, UK; 2 Western General Hospital, Edinburgh, UK; 3 Glasgow Royal Infirmary, Glasgow, UK

**Keywords:** cisplatin, gemcitabine, oesophageal cancer, phase II

## Abstract

Palliative chemotherapy for inoperable/metastatic oesophageal cancer has limited activity. This study assesses the feasibility and activity of gemcitabine and cisplatin in this group of patients. In total, 42 patients with locally advanced/metastatic squamous or adenocarcinoma of the oesophagus were treated with gemcitabine 1250 mg m^−2^ days 1 and 8 and cisplatin 75 mg m^−2^ day 1 in a 21-day cycle. Interim safety analysis was carried out after the first 19 patients suggested significant toxicity. The dose of gemcitabine was subsequently reduced to 1000 mg m^−2^. Patients were assessed for toxicity and response. The median number of treatment cycles per patient was 4 (range 1–6). Grade 3–4 neutropenia occurred in 37% of cycles; however, there was only one episode of neutropenic fever. Nonhaematological toxicities included fatigue, nausea and vomiting. Among 32 patients eligible for response, there were three complete responses and 16 partial responses (overall response rate of 45%); nine patients had stable disease. Median survival was 11 months. The response rate appears to be greatest in those with squamous carcinoma compared to adenocarcinoma (71 *vs* 33%, *P*=0.036). The combination of gemcitabine and cisplatin in this schedule has manageable toxicity and significant activity in patients with locally advanced/metastatic oesophageal cancer and is worthy of further study.

Oesophageal cancer is the ninth most common cancer worldwide. In the UK, the incidence ranges from 13 to 17 per 100 000 for male and around six per 100 000 for female subjects. It is locally advanced/unresectable or metastatic at the time of diagnosis in around 80% patients. Of these, approximately 80% will die within 12 months from diagnosis. The overall 5-year survival is 5%. Over the past 15 years, the incidence of adenocarcinoma of the oesophagus has increased, while that of squamous carcinoma has reduced. It is, however, unclear whether a difference exists in the natural history, sensitivity to treatment or prognosis between these.

Two of the most active agents in single agent phase II trials are cisplatin and 5-fluorouracil (5-FU) (Alberts *et al*, 1992; Bleiberg *et al*, 1997). In combination, these have a response rate of 25–35% in locally advanced or metastatic squamous cell carcinoma of the oesophagus (Bleiberg *et al*, 1997). Despite this activity, there is a pressing need to develop new regimens to improve the response and lessen the toxicity of treatment, thereby optimising quality of life in these patients treated with palliative intent.

Gemcitabine (2′, 2′-difluoro-2′-deoxycytidine) is a nucleoside analogue, which inhibits ribonuclease reductase. Its triphosphate metabolite is also incorporated into DNA resulting in premature termination of replicating DNA strands. The major dose-limiting toxicity of gemcitabine is myelosuppression (Abbruzzese *et al*, 1991); however, this is rarely complicated. Gemcitabine is active against a broad range of tumour types including non-small-cell lung cancer (NSCLC) (Anderson *et al*, 1994), small-cell lung cancer (Cornier *et al*, 1994), breast cancer (Possinger, 1995), head and neck cancer (Catimel *et al*, 1994), pancreatic cancer (Burris *et al*, 1997), bladder cancer (Stadler *et al*, 1997) and ovarian cancer (Lund *et al*, 1994).

In preclinical models, gemcitabine reduces resistance to cisplatin and exhibits synergy with cisplatin (Braakhuis *et al*, 1995; Rose *et al*, 2003). This appears to be sequence dependent (Peters *et al*, 1995; Bergman *et al*, 1996; Crul *et al*, 2003); however, there is no consensus on the optimal schedule *in vivo*. The combination of gemcitabine and cisplatin has been evaluated in NSCLC (Steward *et al*, 1996; Rinaldi *et al*, 2000; Huisman *et al*, 2001; Rossi *et al*, 2002; Soto Parra *et al*, 2002). The cisplatin dose ranges from 50 mg m^−2^ on days 1 and 8 to 100 mg m^−2^ given on day 1 or 2. The gemcitabine dose ranges from 800 to 1250 mg m^−2^ weekly for 2 out of 3 or 3 out of 4 weeks. In NSCLC, the use of a 3 weekly regimen results in significantly fewer dose delays and reductions (19 *vs* 51%), improved dose intensity, similar response rates and reduced grade 3/4 thrombocytopenia (6 *vs* 30%) than a 4 weekly one. This combination has also been tested in ovarian cancer (Nogue *et al*, 2002), transitional cell carcinoma (Lorusso *et al*, 2000) and pancreatic cancer. In the latter, a response rate of 11% was observed with stable disease in 57% (Heinemann *et al*, 2000). The major toxicities are haematological with grade 3/4 leucopoenia in approximately 30% and thrombocytopenia in 5–30% patients. The most frequent nonhaematological toxicities are asthenia, nausea and vomiting and occasional alopecia. Minor degrees of neurotoxicity and transient disturbances in renal function are occasionally observed.

In view of the synergy between these two drugs and the manageable toxicity of the combination, this trial was undertaken to assess its activity in oesophageal carcinoma.

## PATIENTS AND METHODS

### Ethics

Ethical approval was granted by the local research ethics committee in each of the participating centres.

### Eligibility

Patients with locally advanced, unresectable or metastatic, histologically proven, squamous or adenocarcinoma of the oesophagus with bidimensionally measurable disease, ECOG performance status 0–2, life expectancy of >3 months, adequate liver, kidney and bone marrow function were eligible. Prior chemotherapy for metastatic disease was not allowed, although patients who had received neoadjuvant or adjuvant therapy were considered eligible if this had been completed more than 12 months prior to study entry. Patients with a previous history of malignancy with the exception of *in situ* carcinoma of the uterine cervix or nonmelanotic skin cancer were excluded. All participants gave written informed consent before entry.

### Patient assessment and response evaluation

Pretreatment evaluation included a full medical history and examination, full blood count and biochemical profile including urea, electrolytes, and liver function tests and baseline radiological disease assessment. Response was assessed according to the World Health Organisation Criteria. To be evaluable for response, patients had to complete at least three cycles of chemotherapy; however, response rates are calculated by intention to treat. All patients receiving chemotherapy were included in the safety analysis. Toxicity was graded according to the National Cancer Institute common toxicity criteria version 2.

### Treatment

Cisplatin 75 mg m^−2^ in 1 l normal saline over 4 h with pre- and posthydration was given on day 1. Gemcitabine was given at a dose of 1250 mg m^−2^ in 250 ml normal saline over 30 min on days 1 and 8. Treatment was repeated every 21 days. After interim analysis of the first 19 patients, the gemcitabine dose was reduced to 1000 mg m^−2^. Antiemetics were administered according to standard practice at each of the participating centres.

### Dose modification

For a calculated creatinine clearance of 40–60 ml min^−1^, the dose of cisplatin was split and administered on two consecutive days. Cisplatin was omitted if the calculated creatinine clearance was less than 40 ml min^−1^. Cisplatin was discontinued in the event of grade 3 or worse peripheral neuropathy. Following interim analysis, any patient experiencing other non-haematological toxicity (with the exception of alopecia and inadequately controlled nausea and vomiting) of grade 3 or above had a dose reduction of 25% in subsequent cycles. Modifications for haematological toxicity are shown Table 1.

### Statistics

Using Flemings one stage design, 48 patients were required to detect a response rate of 40–60% with a power of 80% at the 5% significance level. The Kaplan–Meier survival curves were generated using SPSS for Windows version 11.5 (Lead Technologies Inc., Charlotte N.C.). Following initial analysis of toxicity in the first 19 patients, it was decided to reduce the total number of patients recruited to 42 as this would not significantly affect the power of the trial, but would have reduced the number of patients exposed to significant toxicity if the toxicity profile was not improved in the second cohort of patients.

## RESULTS

### Demographic data

Between March 2000 and August 2003, 42 patients were recruited; one from Glasgow, 16 from Edinburgh and 25 from Belfast. Patient characteristics are shown in Table 2.

An interim analysis was performed after the first 19 patients were enrolled due to concerns over the level of toxicity. This resulted in a reduction of gemcitabine dose as described previously.

A total of 182 cycles of chemotherapy were completed. All patients received at least one dose of chemotherapy and were therefore eligible for the safety analysis. The median number of cycles received was 4 with a range of 1–6. For the first 19 patients, the median number of cycles was 4; seven patients (37%) completed six cycles and six (32%) patients withdrew due to toxicity. Following amendment of the gemcitabine dose, the median number of cycles was six; 13 patients (57%) completed six cycles and three (13%) patients withdrew due to toxicity. There was no significant difference in the delivered dose intensity pre- and postamendment.

There was significant haematological toxicity prior to the amendment. This was by the reduction of gemcitabine dose to 1000 mg m^−2^. Haematological toxicity is summarised in Table 3. There was one episode of neutropenic fever.

The major non-haematological toxicities were fatigue, nausea and vomiting. Grade 3–4 fatigue occurred in 13 patients (31%). Grade 3–4 nausea and vomiting occurred in seven patients (37%) and 13% of administered treatment cycles preamendment and three patients (13%) and 3% of treatment cycles postamendment. One patient experienced treatment related renal impairment. Grade 1–2 peripheral neuropathy occurred in 10 patients, and grade 1 tinnitus in five patients. Non-haematologic toxicity is summarised in Table 4.

Two patents suffered cerebrovascular accidents and one patient experienced a subendocardial infarct. There were two episodes each of haematemesis and melaena; one such patient died of a GI bleed despite aggressive resuscitation.

**Figure 1:**
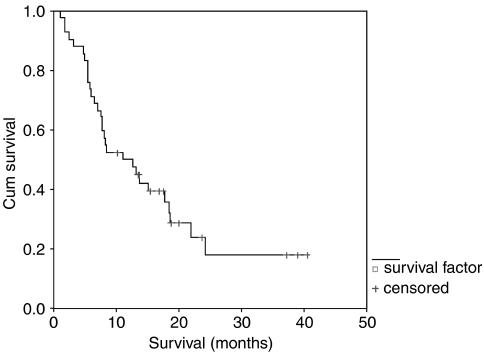
Overall survival.

A total of 32 patients were evaluable for response. Three were unevaluable because they had clinical progression after two cycles, three withdrew early due to toxicity. One patient died of a GI haemorrhage after cycle 1. Two patients suffered cerebrovascular accidents and one a subendocardial infarct. Three complete responses (7%) and 16 partial responses (38%) were observed giving an overall response rate of 45% by intention to treat. The response rate among evaluable patients was 59%. Nine patients (21%) had stable disease and four (10%) progressed on treatment. Median survival was 11 months (95% CI 4.8–17.3 months). (Figure 1).

Three patients recruited to the study had locally advanced inoperable disease. All three were rendered operable by chemotherapy and are still alive at 47, 44 and 30 months from the commencement of chemotherapy. The three patients with a complete response all had squamous carcinoma. The response rate for squamous carcinoma is significantly higher than for adenocarcinoma (71 *vs* 33%, *P*=0.036 Fishers exact test).

Following treatment failure, five patients received further chemotherapy treatment. One patient received a combination of irinotecan with 5-FU and folinic acid and the others received a combination of 5-FU, doxorubicin and mitomycin-c.

## DISCUSSION

The prognosis of oesophageal cancer remains poor. In patients with advanced/unresectable or metastatic disease, response rates of 25–35% are achieved with cisplatin/5FU; however, responses are short-lived and translate into only a small improvement in survival.

Since this study commenced, several trials have examined gemcitabine in the treatment of advanced oesophageal cancer. A phase II trial of single agent gemcitabine in metastatic oesophageal cancer showed no activity in the 21 patients treated (Sandler *et al*, 2000). The Southwest Oncology Group conducted a multicentre phase II trial of gemcitabine 1000 mg m^−2^ on days 1, 8 and 15, and cisplatin 100 mg m^−2^ on day 15 of a 28-day cycle in 64 patients with advanced oesophageal cancer. Treatment was well tolerated, with grade 3/4 neutropenia reported in 31% patients. Median survival was 7.3 months with a 1-year survival of 20% (Urba *et al*, 2004).

A further phase II study used cisplatin 50 mg m^−2^ on days 1 and 8 followed by gemcitabine 800 mg m^−2^ on days 2, 9 and 16 every 28 days in 36 patients with advanced oesophageal cancer. This was myelosuppressive with grade ⩾3 neutropenia in 83% patients (three cases of neutropenic fever) and grade 3/4 thrombocytopenia in 67% patients. Myelotoxicity was cumulative requiring omission of gemcitabine on day 16 in 61% cases. A response rate of 41% was observed with a median survival of 9.8 months. Response rates and median survivals were comparable for squamous and adenocarcinomas (Kroep *et al*, 2004). In a phase II study of 40 chemonaïve patients with advanced/metastatic gastric cancer, this schedule resulted in grade 3/4 leucopenia and thrombocytopenia in 58 and 48% of patients, respectively, with omission of gemcitabine on day 16 in 55% cycles. The response rate was 30% with a median survival of 11 months (De Lange *et al*, 2004).

Our study, similarly, demonstrates the activity of gemcitabine and cisplatin in advanced oesophageal cancer. The overall response rate of 45% and the median overall survival of 11 months are similar to other trials of this combination. The initial dose and schedule of gemcitabine 1250 mg m^−2^ with cisplatin 75 mg m^−2^ was based on reports of the tolerability and activity in NSCLC (Castellano *et al*, 1998). This regimen, however, results in unacceptable toxicity for patients with oesophageal cancer. With a gemcitabine dose of 1000 mg m^−2^ on days 1 and 8 combined with cisplatin 75 mg m^−2^ on day 1 of a 21-day cycle, toxicity is less than that reported using more intensive schedules of administration. This is consistent with a previous schedule-finding study, which demonstrated that leucopoenia was schedule dependent and less common when gemcitabine was given prior to cisplatin (Kroep *et al*, 1999).

Three cerebro/cardiovascular events occurred on study. This finding is of concern, however it should be noted that shared risk factors make cerebro- and cardiovascular disease common comorbidities in patients with oesophageal cancer and, as this is a small study, no conclusions can be drawn.

In those with squamous carcinoma, the response rate was 71 *vs* 33% for adenocarcinoma. Although numbers are small, this difference is statistically significant. This did not translate into any survival difference. Previous phase II trials of cisplatin-based regimens have suggested that the response rate for squamous tumours is slightly higher than for adenocarcinoma (Ajani, 1994). In those receiving neoadjuvant chemoradiotherapy for oesophageal cancer, the complete pathological response rate has been reported to be higher for squamous carcinoma than for adenocarcinoma (44.4 *vs* 35.5%); however, 5-year survival rates in these complete responders is not significantly different Makary *et al*, 2003). Previous trials of gemcitabine and cisplatin in oesophageal carcinoma have not demonstrated any difference in response or survival by histological subtype (Kroep *et al*, 2004). The activity of this combination is comparable to other platinum- or taxane-based doublets (Ilson *et al*, 2000; Jatoi *et al*, 2002).

Our study and other recently reported trials suggest that the combination of gemcitabine and cisplatin in oesophageal cancer is active and worthy of further study in randomised controlled trials. Although the response rate is not superior to other cisplatin-based regimens, we have employed a straightforward schedule reducing the need for hospital admission for chemotherapy administration and the need for central venous access devices for the delivery of infusional 5-FU.

## Figures and Tables

**Table 1:** Dose modifications for haematological toxicity

**Days**	**ANC**		**Plts**	**% Full dose**
*Preamendment*				
1 and 8	⩾1 × 10^9^ l^−1^	AND	>100 × 10^9^ l^−1^	100
1 and 8	0.5–0.9 × 10^9^ l^−1^	OR	50–99 × 10^9^ l^−1^	50
1 and 8	<0.5 × 10^9^ l^−1^	OR	<50 × 10^9^ l^−1^	Nil
				
*Postamendment*				
1	⩾1.5 × 10^9^ l^−1^	AND	>100 × 10^9^ l^−1^	100
1	<1.5 × 10^9^ l^−1^	OR	<100 × 10^9^ l^−1^	Delay until recovery
8	⩾1.5 × 10^9^ l^−1^	AND	>100 × 10^9^ l^−1^	100
8	1–1.5 × 10^9^ l^−1^	OR	50–100 × 10^9^ l^−1^	50
8	<1 × 10^9^ l^−1^	OR	<50 × 10^9^ l^−1^	Omit

ANC=absolute neutrophil count; Plts=platelets.

**Table 2:** Demographic data

Median age (range)	60 (37–79) years
Male : female	34 : 8
	
*Performance status*	
0	13
1	22
2	5
Unrecorded	2
	
*Histology*	
Squamous	14
Adeno	27
Mixed	1
	
*Stage*	
Locally advanced	3
Metastatic	39

**Table 3:** Haematological toxicity in all cycles

	**Preamendment (*n*=77 cycles)**		**Postamendment (*n*=105 cycles)**	
	**Grade 3**	**Grade 4**	**Grade 3**	**Grade 4**
	***n* (%)**	***n* (%)**	***n* (%)**	***n* (%)**
Neutropenia	25 (32)	14 (18)	27 (26)	2 (2)
Thrombocytopenia	13 (17)	0	17 (16)	0
Anaemia	1 (1)	0	3 (3)	0

**Table 4:** Major non-haematologic toxicity in all cycles

	**Pre-amendment (*n*=77 cycles)**		**Post-amendment (*n*=105 cycles)**	
	**Grade 2**	**Grade 3/4**	**Grade 2**	**Grade 3/4**
	***n* (%)**	***n* (%)**	***n* (%)**	***n* (%)**
Aesthenia	21 (27)	11 (14)	16 (15)	6 (6)
Diarrhoea	2 (3)	3 (4)	1 (1)	0
Stomatitis	3 (4)		1 (1)	
Nausea/vomiting	11 (14)	10 (13)	8 (8)	3 (3)
